# The prevalence of Early Childhood Caries in 1-2 yrs olds in a semi-urban area of Sri Lanka

**DOI:** 10.1186/1756-0500-4-336

**Published:** 2011-09-09

**Authors:** Shanika LM Kumarihamy, Lushanika D Subasinghe, Prasanna Jayasekara, Sanjeewa M Kularatna, Priyaka D Palipana

**Affiliations:** 1 Faculty of Dental Sciences, University of Peradeniya, Peradeniya, Sri Lanka; 2 Dental Institute, Maharagama, Sri Lanka; 3 Office of Deputy Director General (Dental Services), Ministry of Health, Colombo, Sri Lanka

## Abstract

**Background:**

ECC remains a problem in both developed and developing countries and ECC has been considered to be present in epidemic proportions in the developing countries. The aetiology and associated factors of ECC should be studied adequately to overcome this health hazard. The objective of this study is to determine the prevalence of ECC in 1 to 2 years old children in some selected MOH areas (semi-urban) in the district of Colombo, Sri Lanka.

**Methods:**

This study was conducted as a cross sectional study. A total of 422 children aged 1-2 years were selected using systematic sampling technique in Maharagama, Piliyandala, Nugegoda and Boralesgamuwa MOH areas in Colombo district, Western province, Sri Lanka. The pre-test was done initially with 10 children aged 1 1/2 year olds.

Prior to the clinical examination of each child, a questionnaire consisting questions regarding tooth brushing, dietary habits, breast and bottle feeding, long term medications(Sweetened medications taken more than 3 months), attending a dental clinic during pregnancy of mother and socio-economical status of the family was administered to mothers of those children. Sterile dental mouth mirrors were used to detect ECC in children.

**Results:**

The prevalence of ECC of the whole sample of 410 children aged 1-2 years was 32.19% and the mean dmft was 2.01 and the mean dmfs was 3.83. From the children who had ECC 95% were untreated. There were significant relationships between dmft and long term use of medications (p < 0.000), intake of sugar with milk (p = 0.013), sweet consumption (p = 0.013), employment of mothers (p < 0.000) and visiting a dental clinic during pregnancy (p < 0.000).

**Conclusions:**

This study documents high prevalence and severity of ECC among 1-2 years old children in four selected MOH areas of Colombo district and caries in most of the children with ECC (95%) were untreated. Results reveal an urgent need to increase awareness among the public about ECC and institute preventive strategies.

## Background

Early childhood caries (ECC) is a widespread condition seen among children throughout the world. According to American Academy of Paediatric Dentistry ECC can be defined as the presence of one or more primary teeth with caries (cavitated or non-cavitated) in a child 71 months of age or younger.

Many deciduous teeth are lost due to ECC, but importance of deciduous teeth cannot be neglected as they are essential for a healthy permanent dentition, proper nutrition and beauty [1]. Moreover, inability to produce labio-dental fricatives and dental fricatives leads the child to develop incorrect language patterns. Untreated ECC can lead to abscesses, pain and malocclusion. High ECC prevalence in the children results in disturbance in education and parents' absenteeism to work. The cost of treatment is also a burden to a country like Sri Lanka which offers health facilities free of charge. Hence forth the aetiology and associated factors of ECC should be studied adequately to overcome this health hazard.

The aetiology of ECC is complex and multifactorial [2]. It is a plaque induced infectious disease caused by endogenous bacteria [3]. Parents and caregivers are responsible for transmitting Streptococci mainly *Streptococcus mutans *to the child's oral cavity, which is free of them at birth [4,5]. Presence of fermentable carbohydrates and harmful plaque bacteria in combination results in acid production and demineralization of the enamel [6]. The tooth is more susceptible to ECC, in the period immediately after eruption and prior to final maturation, at the time the child is breast fed.

Breast milk may promote tooth decay [7], especially when the baby is at breast throughout the night. Bottle feeding and giving pacifiers dipped in sweeteners will also promote ECC [8]. In chronic disease conditions children have to follow up certain medications that increase the risk of ECC (E.g.:-Antihistamines like chlorphenamine maleate). Because of the stressful lifestyle of parents, less attention is paid on child's teeth. Studies have shown that poor socio-economical status also increase the incidence of having ECC [9-12].

ECC remains a problem in both developed and developing countries. ECC has been considered to be present in epidemic proportions in the developing countries. Numerous studies have been conducted to find the prevalence of ECC. In England and USA the prevalence is reported to be 6.8 -12% and 11-53.1% respectively [13]. A comprehensive review of the occurrence of the caries on maxillary anterior teeth in children, including numerous studies from Europe, Africa, Asia, the Middle East, and North America, found the highest caries prevalence in Africa and South-East Asia [13]. In India a prevalence of 44% has been reported for caries in 8- to 48-month-olds [14]. T. Vachirarojpisan (2004) has shown prevalence of caries is 57.5% and 82.8% in 11-14 month old children and in 15-19 month old Thai children [15].

There are only few studies and published reports on ECC of 1 to 2 yrs olds in Sri Lanka. One such study revealed that the prevalence percentage of ECC in 1-2 year olds is 23% [16] and the National oral health survey 2002/03 reported a prevalence of 65% among 5 year olds [17]. Hence there is a steady rise in ECC from 1 year to 5 years. To reduce the ECC prevalence in Colombo district, which is the most populated district in the country, data specific to Colombo should be obtained.

### Objective

The objective of this study is to determine the prevalence of ECC in 1 to 2 yrs olds in some selected MOH areas (semi-urban) in the district of Colombo, Sri Lanka.

## Methods

Initially only 1 1/2 years old children at vaccination centres were planned to include in the study. Due to the difficulty in finding enough children specifically aged 1 1/2 years which was encountered during the pre-test, it was decided to take one to two years old children present at both vaccination and weighing centres in Maharagama, Piliyandala, Nugegoda and Boralesgamuwa MOH areas in Colombo district, Western province, Sri Lanka.

Taking into consideration of the non availability of previous data, the prevalence was taken as 50%. By adding 10% for the non-respondents, the sample size was taken as 422. It was encountered from the pre-test that around 50 children attend a centre a day. The centres were visited according to the alphabatical order. Every 3^rd ^child who visited the centre was selected for the sample and given an appointment for examination. Since the mothers of 12 children did not give their consent on the appointment date, the final sample size was 410 children.

Children in whom at least one tooth has erupted were included in the study. Consent was obtained from mothers. Prior to the clinical examination of each child, a questionnaire consisting questions regarding tooth brushing, dietary habits, breast and bottle feeding, giving pacifiers, long term medications, attending to a dental clinic during pregnancy of the mother and socio-economical status of the family was administered to mothers of those children.

Ethical approval was obtained from the Ethical Board, Postgraduate institute of medicine; University of Colombo, Sri Lanka. Permission for the study was obtained by Deputy Director General of Dental Services, Ministry of Health, Sri Lanka and Regional Director of Health Services, Colombo, Sri Lanka. The pre-test was done initially with 10 children aged 1 1/2 year olds at Maharagama MOH.

### Clinical examination

All examinations were conducted by the researchers, two undergraduates from the Faculty of Dental Sciences, University of Peradeniya, Sri Lanka, after a two weeks training programme under the Dental Public Health Consultant at Dental Institute, Maharagama. The examiners were especially trained for identifying non cavitated carious lesions (white spot lesions). Examiner calibration was conducted before the survey. Kappa scores higher than 0.9 were attained for both inter- and intra-examiner calibration exercises for identifying cavitated and non-cavitated carious lesions indicating high reliability between investigators. The children were examined after a brief self-introduction by the examiners. Sterile dental mouth mirrors were used to detect ECC in children. The tooth surfaces were cleaned and dried using cotton wool and were examined under the natural light [18]. Findings were recorded according to WHO criteria for caries [19]. Measures were taken to minimize intra and inter examiner variations. The children with untreated carious lesions were referred to the Dental Institute, Maharagama.

Data Analysis was undertaken using the Statistical Package for Social Science (SPSS version 16). Significance was set at P < 0.05 (Significance level 95%). Mean dmft and standard deviation of various groups were calculated. The mean difference of dmft between groups was compared with ANOVA with 95% significance.

## Results

The prevalence of ECC of the whole sample of 410 children aged 1-2 years was 32.19% and the mean dmft was 2.01 and the mean dmfs was 3.83. From the children who had ECC 95% were untreated.

Table 1 shows the relationship between ECC and some socio-demographic factors. The caries prevalence was higher in 18-24 month olds compared to 12-18 month olds. In considering MOH areas the mean dmft was highest in Boralesgamuwa and was lowest in Maharagama. The prevalence of ECC was highest in children in Nugegoda MOH area (74.07%) followed by Maharagama (58.06%) and Piliyandala (50.98%). The lowest ECC prevalence was encountered in Boralesgamuwa MOH area (48.84%). Employment of mothers was strongly related to ECC. Monthly family income was not related to dmft, but highest mean dmft was reported in middle income families.

**Table 1:** ECC and some socio-demographic factors

	%	Mean dmft(sd)	P value	F	df
**Age in months**					
12-18	68	1.37(2.28)	0.000	43.49	1
> 18-24	32	3.35(3.75)			
**MOH Area**					
Boralesgamuwa	42.0	2.24(3.11)	0.000	13.73	4
Nugegoda	7.6	1.42(2.19)			
Maharagama	13.1	1.09(2.21)			
Piliyandala	37.3	2.18(3.14)			
**Mothers employment**					
Mothers with jobs	15.1	0.63(3.21)	0.000	22.94	2
Mothers without jobs	84.9	2.07(2.93)			
**Monthly family income in Rs**.					
< 10000	17.8	1.52(2.49)	0.102	2.08	3
10000-30000	64.6	2.28(3.19)			
> 30000	17.6	1.50(2.43)			

Table 2 shows that there were significant relationships between dmft and long term use of medications (p < 0.000), intake of sugar with milk (p = 0.013), sweet consumption (p = 0.013) and mothers visiting a dental clinic during pregnancy (p < 0.000).

**Table 2:** The relationship between ECC and some related factors

	%	Mean dmft(sd)	P value	F	df
**Long term medications**-yes	15.36	2.02(3.67)			
no	84.63	2.00(2.84)	0.000	22.16	2
**Night feeding**-yes	83.2	1.95(2.80)	0.352	0.86	1
No	16.8	2.34(3.87)			
**Bottle feeding**-yes	54	1.94(2.04)	0.614	0.25	2
No	46	2.09(2.89)			
**Sugar intake with milk**-yes	74.7	2.27(3.12)	0.013	6.21	1
no	25.3	1.50(2.61)			
**Sweet consumption**					
< 3 days/week	55.8	1.71(2.79)	0.013	4.02	3
3-4	20.1	2.36(3.45)			
> 4	24.1	2.79(3.01)			
**Snacking**					
< 3 days/week	19.1	1.36(2.55)	0.013	1.53	3
3-4	19.1	2.04(3.07)			
> 4	68.1	2.19(3.04)			
**Average sugar added to food and beverages per day**					
< 3 teaspoons	85.2	1.09(2.83)	0.181	1.99	5
> 3	14.8				
**Frequency of teeth cleaning -**					
≤1	32.93	1.75(2.98)	0.176	1.17	6
≥2	67.07	2.08(2.95)			
**Dentifrice-**					
none	23.1	1.44(2.47)	0.222	3.86	2
Fluoridated	47.1	2.39(3.23)			
Non-fluoridated	29.8	2.11(3.06)			
**Mothers visited a dental clinic during pregnancy-**					
yes	70.73	2.11(3.01)	0.000	23.00	2
no	29.26	1.75(2.90)			

In considering prevalence of ECC by teeth affected, deciduous upper central incisors were the most affected teeth followed by deciduous upper lateral incisors. When considering the surface patterns affected by caries, upper labial surfaces of teeth (31.6%) were the most affected followed by the mesial surfaces of upper teeth (16.8%) while lingual surfaces of lower teeth (1.01%) were the least affected (Figure).

The table 3 shows the five tooth surfaces mostly affected by caries. According to that the labial surface of deciduous upper right central incisor was the most affected tooth surface by ECC.

**Table 3:** Tooth surfaces most affected by caries

Surface	Percentage of children affected by caries
URA -labial surface	35.85
ULA -labial surface	34.63
URB -labial surface	29.02
ULB -labial surface	28.29
ULA -mesial surface	20

## Discussion

This study documents one of the major oral health issues in early childhood. In considering the decayed teeth, labial surfaces of deciduous maxillary central incisors were the most affected. This may be due to the milk pooling between the upper lip & deciduous maxillary central incisor when children are fed at night and there is reduced salivary flow when sleeping [20]. In contrast lower anteriors were the least affected because tongue and lower lip covers them.

The sample of 410 children were categorized into 2 groups based on age; 12-18 months and > 18-24 months as the first primary teeth erupt around 5-6 months after birth, and since nursing, dietary, and tooth brushing habits change as the child becomes older. Caries prevalence was relatively higher in 18-24 months old aged group compared to 12-18 months old children, may be due to adopting adverse dietary habits when growing older and increased length of time the teeth were exposed to cariogenic food. Reduction in frequency and ceasing of breast feeding, high frequency of snacking and sweet consumption, increased consumption of milk with added sugar and cariogenic solid food may be some of those contributory adverse dietary habits.

High contrast of the presence of ECC among the 4 MOH areas suggests that there is a requirement to conduct a proper standard programme in order to combat the problem of ECC in all areas.

Though it has been shown in the scientific literature that is ECC related to night feeding, snacking, average sugar intake per day, using of fluoridated toothpaste, frequency of tooth cleaning and socio-economic level of the family [13,21-24], such relationships were not found in the present study. The reason for the fact that night feeding was not related to ECC may be the high awareness of mothers regarding precautions that should be taken to prevent pooling of milk in mouth after feeding.

There is a strong relationship between long term medications and ECC. This may be due to the effect that the medications were sweetened and also teeth cleaning habits are neglected during ill-health. So health care providers should be encouraged not to prescribe sweetened medications and legislations must be administered to ban on importing and selling them.

It is interesting to find that children of the mothers who visited a dental clinic during pregnancy had a higher percentage of ECC compared to children whose mothers who had not visited a dental clinic during pregnancy. This may be due to the fact that, mothers who had dental problems had a more chance of visiting a dental clinic than who hadn't visited a clinic. Microflora responsible for caries is transmitted to the child's oral cavity by care givers mainly from the mother due to tasting food, kissing and sharing utensils. The reason for low ECC in children whose mothers are employed may be due to the high level of education and their awareness about ECC.

One of the main risk factors for high prevalence of ECC was unhealthy dietary habits; cariogenic foods like biscuits been easily accessible and most available economical food for parents may be a major contributor. Since adequate education on ECC is not provided to the community, more cariogenic food is given to children on demand. The school dental clinics in Sri Lanka which are occupied by school dental therapists are meant only for children between 3-13 years and there is no properly organized oral health programme catering children below age 3. Since the cost of dental treatment in the private sector is also considerably high, parents are reluctant to take their children for private dental clinics. These factors may also contribute to this high ECC prevalence of aged 1-2 year old children. Henceforth ECC is a health problem that needs attention and resources of the community.

Screening for dental caries should start as soon as the first primary tooth erupts or not later than one year of age. Oral health programmes should be established focusing on mothers, caregivers, community health workers, preschool teachers and children. Further epidemiological data must be gathered through surveys in other regions of the country to support oral health programmes. It also needs to raise the awareness on the diagnosis prevention and treatment of ECC among health care workers including paediatricians, physicians, nurses and midwives. Especially facilities for preventive dental work (Fluoride varnish/gel application, fissure sealants) should be enhanced. Mothers should be educated regarding recognition of early signs of ECC, proper nutrition, supervise tooth brushing, cautious use of fluoride sources in high carious risk children and taking children to a dental clinic at the end of the 1^st ^year [25].

The limitations of this study are not using of random sampling method to select the sample and the difficulties encountered in co-operating and co-ordinating with MOH staff in gathering children and transportation. Out of most of the relationships, age and eruption pattern of teeth can be two of the major co-founding factors that affect the results.

## Conclusions

This study documents high prevalence and severity of ECC among 1-2 years old children in four selected MOH areas of Colombo district. And caries in most of the children with ECC (95%) were untreated. Results reveal an urgent need to increase awareness among the public about ECC and institute preventive strategies and standard programmes to control ECC in every MOH area.

## Abbreviations

ECC: Early Childhood caries; MOH: Medical Office of Health; dmft: decayed, missing, filled teeth; dmfs: decayed, missing, filled surfaces of teeth; sd: standard deviation; df: degree of freedom; URA: Deciduous upper right central incisor; ULA: Deciduous upper left central incisor; URB: Deciduous upper right lateral incisor; ULB: Deciduous upper left lateral incisor

## Competing interests

The authors declare that they have no competing interests.

## Authors' contributions

**SLMK**. Has made substantial contributions to conception and design, acquisition of data, analysis and interpretation of data; has been involved in drafting the manuscript and has given final approval of the version to be published. **LDS**. Has made substantial contributions to design, acquisition of data, analysis and interpretation of data; has been involved in drafting the manuscript and has given final approval of the version to be published. **PJ**. has made substantial contributions to conception and design, acquisition of data, has given final approval of the version to be published. **SMK**. has made substantial contributions to analysis and interpretation of data and has given final approval of the version to be published. **PDP**. has made substantial contributions to conception and design, acquisition of data, and has given final approval of the version to be published.

**Figure 1:**
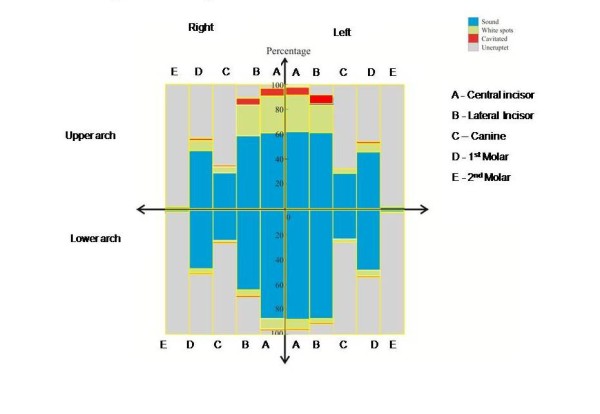
**The caries pattern according to teeth**. Deciduous upper central incisors were the most affected teeth by caries activity followed by deciduous upper lateral incisors.
